# Scenario-Based Ethical Reasoning Among Healthcare Trainees and Practitioners: Evidence from Dental and Medical Cohorts in Romania

**DOI:** 10.3390/healthcare13202583

**Published:** 2025-10-14

**Authors:** George-Dumitru Constantin, Bogdan Hoinoiu, Ioana Veja, Ioana Elena Lile, Crisanta-Alina Mazilescu, Ruxandra Elena Luca, Ioana Roxana Munteanu, Roxana Oancea

**Affiliations:** 1 Discipline of Clinical Skills, Department I Nursing, “Victor Babes” University of Medicine and Pharmacy Timisoara, 300041 Timisoara, Romania; george.constantin@umft.ro; 2 Doctoral School, “Victor Babes” University of Medicine and Pharmacy Timisoara, Eftimie Murgu Square 2, 300041 Timisoara, Romania; 3 University Clinic of Oral Rehabilitation and Dental Emergencies, Faculty of Dentistry, “Victor Babes” University of Medicine and Pharmacy Timisoara, Eftimie Murgu Square No. 2, 300041 Timisoara, Romania; hoinoiu@umft.ro (B.H.); luca.ruxandra@umft.ro (R.E.L.); munteanu.roxana@umft.ro (I.R.M.); 4 Interdisciplinary Research Center for Dental Medical Research, Lasers and Innovative Technologies, Revolutiei 1989 Avenue No. 9, 300070 Timisoara, Romania; 5 Department of Dental Medicine, Faculty of Dentistry, “Vasile Goldis” Western University of Arad, 310025 Arad, Romania; 6 Teacher Training Department, Politehnica University Timisoara, 300596 Timisoara, Romania; alina.mazilescu@upt.ro; 7 Translational and Experimental Clinical Research Centre in Oral Health, Department of Preventive, Community Dentistry and Oral Health, Faculty of Dental Medicine, “Victor Babes” University of Medicine and Pharmacy Timisoara, 300041 Timisoara, Romania; roancea@umft.ro

**Keywords:** ethical dilemmas, scenario-based dilemmas, decision-making, medicine, dentistry, Romania

## Abstract

**Background and Objectives:** Clinical ethical judgments are often elicited through scenario-based (vignette-based) dilemmas that guide interpretation, reasoning, and moral judgment. Despite its importance, little is known about how healthcare professionals and students respond to such scenario-based dilemmas in Eastern European settings. This study explored differences in ethical decision-making between senior medical/dental students and practicing clinicians in Romania, focusing on how scenarios-based dilemmas influence conditional versus categorical responses. **Materials and Methods:** A cross-sectional survey was conducted with 244 participants (51 senior students; 193 practitioners). Respondents completed a validated 35-item questionnaire presenting hypothetical ethical scenarios across seven domains: informed consent, confidentiality, medical errors, public health duties, end-of-life decisions, professional boundaries, and crisis ethics. Each scenario used a Yes/No/It depends response structure. Group comparisons were analyzed using chi-square and non-parametric tests (α = 0.05). **Results:** Scenario-based dilemmas elicited frequent conditional reasoning, with “It depends” emerging as the most common response (47.8%). Strong consensus appeared in rejecting concealment of harmful errors and in treating unvaccinated families, reflecting robust professional norms. Divergences arose in areas where scenario-based dilemmas emphasized system-level duties: students more often supported annual influenza vaccination (52.9% vs. 32.6%, *p* = 0.028) and organ purchase authorization (76.47% vs. 62. 18%, *p* = 0.043), while practitioners more frequently endorsed higher insurance contributions for unhealthy lifestyles (48.7% vs. 23.5%, *p* = 0.003). **Conclusions:** Scenario-based dilemmas strongly shape moral decision-making in healthcare. While students tended toward principle-driven transparency, practitioners showed pragmatic orientations linked to experience and system stewardship. To promote high-quality clinical work and align decision-making with best practice and health policy, our findings support institutional protocols for transparent error disclosure, continuing professional development in ethical communication, the possible adoption of annual influenza vaccination policies for healthcare personnel as policy options rather than categorical imperatives, and structured triage frameworks during crisis situations. These proposals highlight how scenario-based ethics training can strengthen both individual reasoning and systemic resilience.

## 1. Introduction

Medical ethics provides the foundational framework guiding physician behavior, grounded in the principles of beneficence, non-maleficence, respect for autonomy, and justice [1,2]. These cardinal values underpin clinical decision-making and safeguard both patient welfare and professional integrity. In everyday practice, however, ethical dilemmas are pervasive-ranging from resource allocation within constrained systems, end-of-life care, informed consent, confidentiality breaches, and conflicts of interest to responding to medical errors [3,4,5]. Physicians must often balance competing principles while navigating legal, institutional, and cultural influences [6]. Beyond mapping these dilemmas, this study explicitly considers how such decisions reverberate for patients (e.g., safety, trust, and shared decision-making) and for the medical profession (e.g., professional identity, legal exposure, and public confidence) [1,2,3,4,5,6].

Recent global developments, such as the COVID-19 pandemic, have intensified the moral challenges faced by medical professionals, necessitating difficult triaging decisions, prioritization of scarce resources, and protecting both patients and staff under distressing conditions [7,8,9]. These challenges have also stimulated broader discussions about preparedness, professional resilience, and the integration of ethical reasoning into healthcare curricula [10,11,12,13]. Even outside crisis scenarios, routine practice presents nuanced ethical conflicts, such as how much information to disclose to patients, managing pharmaceutical pressures, and reconciling personal beliefs with professional responsibilities.

Ethical reasoning is shaped not only by theoretical training but also by accumulated clinical experience and cultural context [14,15]. Medical students, immersed in academic instruction, frequently approach dilemmas from idealistic or normative standpoints. In contrast, practicing clinicians confront ethical decisions within real-world constraints, balancing ethical theory with institutional policies, economic realities, and patient psychosocial needs [16,17,18]. These differing vantage points, while not contradictory, offer complementary insights into the practical application of medical ethics.

Despite its significance, comparative empirical research on ethical attitudes between medical students and physicians remains limited—especially within Eastern Europe and Romania, where systemic, cultural, and institutional frameworks uniquely shape healthcare practice [19,20]. Existing Western-centric studies may not adequately reflect the Romanian context-characterized by underfunded systems, workforce emigration, and evolving public expectations [21]. Furthermore, bioethics education in Romania, although increasingly present in curricula, varies in depth and integration with clinical training. Students often engage only theoretically with ethics, whereas practicing clinicians may lack continuing professional ethics development [10,22]. Identifying areas of agreement and divergence between these groups can inform curricula enhancements, promote coherence between academic training and practice, and ultimately strengthen ethical performance in healthcare.

Despite extensive international literature on ethical decision-making and scenario-based (vignette-based) dilemmas, most empirical studies have been conducted in Western Europe and North America, with limited attention to Eastern Europe [19,20,21]. Romania provides a distinctive case due to systemic underfunding, physician migration, evolving patient-rights frameworks, and heterogeneous integration of ethics into medical curricula [9,21,22]. Comparative studies show regional challenges such as disparities in legal compliance [9], insufficient physician knowledge of consent and confidentiality [23], and heightened stress and ethical tensions during the COVID-19 pandemic [21,22]. By situating our investigation in this context, the present study complements Western-centric research and shows how scenario-based dilemmas shape ethical reasoning across different career stages in an Eastern European healthcare system.

This study aimed to examine and compare the ethical decision-making of senior medical and dental students versus practicing clinicians in Romania. We hypothesized that students, shaped primarily by theoretical instruction, would show more principle-driven responses, whereas practitioners, influenced by clinical realities, would demonstrate more pragmatic orientations. Using a validated questionnaire covering seven domains of clinical ethics, we sought to identify patterns of consensus and divergence, assess the influence of scenario-based dilemmas on ethical reasoning, and evaluate the implications for ethics education and health policy.

## 2. Materials and Methods

### 2.1. Study Design

The present investigation was conceived as a descriptive and analytical cross-sectional study aimed at exploring how medical students and practicing clinicians in Romania respond to ethically challenging clinical situations. The present investigation was conceived as a descriptive and analytical cross-sectional study, guided by the STROBE (Strengthening the Reporting of Observational Studies in Epidemiology) statement to ensure methodological transparency and reproducibility. In line with these guidelines, we provided detailed reporting of study design, participant selection, variables, and statistical analyses to enhance clarity and comparability with similar observational research.

The study sought to capture the nuanced interplay between theoretical ethical instruction, which predominates during medical training, and the pragmatic, experience-informed decision-making that develops during years of clinical practice. By examining a diverse array of ethically sensitive scenarios, the study aimed to identify both areas of convergence and points of divergence between these two groups.

### 2.2. Participants and Recruitment

A total of 244 individuals participated, divided into two cohorts. The student cohort comprised 51 senior students (years 4–6) from the “Victor Babes” University of Medicine and Pharmacy Timisoara, Timișoara (General Medicine 17.65%; Dentistry 82.35%; 84.31% female). The physician cohort included 193 graduates actively engaged in clinical practice in Romania (general practitioners 31.61%; dentists 68.39%; 76.17% female) with 1–28 years of experience (median 12; IQR 6–21). In Romania, dentistry is legally recognized as a branch of medicine; therefore, dentists and medical doctors are collectively referred to as “clinicians” in this study.

Participants were recruited using a convenience sampling strategy: students were invited during scheduled academic activities; physicians were approached at professional meetings and via targeted electronic invitations. Inclusion criteria required final–three-year enrollment for students (to ensure prior ethics instruction) and active clinical practice for physicians. We excluded first–third-year students, retired physicians, and incomplete questionnaires. Because all participants studied or graduated from the same university, the sample shares a curricular background, which may limit representativeness beyond this institutional context.

### 2.3. Instrumentation

The research instrument was a 35-item questionnaire originally developed and validated by Vincent Richeux and Véronique Duquéroy in French settings, where it has been applied in both medical student and physician cohorts to assess ethical reasoning across clinical dilemmas. For the Romanian adaptation, we followed a forward–backward translation procedure (Romanian–French) conducted by bilingual experts to ensure semantic equivalence. A panel of three medical ethics lecturers reviewed the instrument for cultural and legal appropriateness, and pilot testing with 10 students and 10 physicians confirmed comprehensibility and contextual relevance, leading to minor wording adjustments. Internal consistency in our sample was acceptable to good, with Cronbach’s alpha values ranging from 0.71 to 0.84 across the seven domains.

The scenarios were culturally adapted for Romania through translation, expert review, and pilot testing to ensure contextual relevance. Each item presented a hypothetical but realistic clinical scenario with three possible responses: Yes, No, or It depends. Scenarios covered seven domains: informed consent and transparency; confidentiality and mandatory reporting; medical error and professional responsibility; end-of-life care and allocation of limited resources; external influences and conflicts of interest; moral and cultural norms; and ethics in exceptional circumstances.

To illustrate how scenario-based dilemmas influenced responses, one representative item from each ethical domain is presented herein:

– Informed consent and transparency: “If a patient’s family requests that you withhold the diagnosis of cancer, would you comply?” (Yes/No/It depends).
– Confidentiality and mandatory reporting: “If you suspect child abuse but lack definitive proof, are you obliged to report this case?”
– Medical error and professional responsibility: “If you make a medical error that does not cause harm, should you disclose it to the patient?”
– End-of-life care and resource allocation: “In a situation of scarce resources, should younger patients be given priority over older ones?”
– Conflicts of interest: “Would you accept a non-monetary benefit (e.g., a gift or trip) from a pharmaceutical representative?”
– Moral and cultural norms: “Would a romantic or sexual relationship with a current patient be acceptable under certain circumstances?”
– Ethics in exceptional circumstances: “If protective equipment is insufficient, is it ethically justifiable to abandon care for infectious patients?”

The Yes/No/It depends format was chosen to balance categorical judgments with conditional reasoning, approximating real-world decision-making. While no open-ended comments were collected, the distribution of “It depends” responses served as a proxy for nuanced, context-sensitive moral reasoning across scenarios.

### 2.4. Data Collection

Data collection was carried out over a four-month period, between November 2023 and February 2024. For students, the paper-based questionnaire was distributed during scheduled academic activities, with completion supervised by a member of the research team. For physicians, both paper-based distribution at professional meetings and a secure online version (via Google Forms, password-protected) were employed to maximize accessibility.

Participation was entirely voluntary and uncompensated. All participants received an information sheet outlining the study’s objectives, the anonymous nature of data collection, and their right to withdraw at any stage without consequence. To reduce the influence of social desirability bias, it was emphasized that there were no correct or incorrect answers and that the study was intended to capture personal ethical reasoning rather than test knowledge. Average completion time was approximately 15–20 min.

### 2.5. Ethical Approval and Consent

The study protocol received ethical approval from the “Victor Babes” University of Medicine and Pharmacy Timisoara Ethics Committee, Timișoara, Romania (protocol code No. 87/20.10.2023 rev 2025). Participation was voluntary and anonymous. Before accessing the questionnaire, each participant was required to check an online consent box confirming agreement to participate; this step was mandatory in order to proceed to the survey questions.

### 2.6. Statistical Analysis

Data analysis was performed using IBM SPSS Statistics v.27 (IBM Corp., Armonk, NY, USA). Descriptive statistics were computed for all variables, expressed as absolute frequencies and percentages. Between-group comparisons of categorical responses (students vs. physicians) were performed using Pearson’s Chi-square test (χ^2^). For items with small expected cell counts, Fisher’s exact test was applied. For the overall distribution reported, we computed an aggregate by pooling responses across all 35 items (equal weight per item) within each cohort and overall.

To assess the relationship between ordinal responses and participant age, the Kruskal–Wallis H test was employed. Where significant differences were detected, post hoc pairwise comparisons were conducted using Bonferroni-adjusted *p*-values to control for Type I error. The statistical significance was set at *p* < 0.05. In addition to *p*-values, we reported effect sizes—Cramer’s V for chi-square tests and η^2^ for Kruskal–Wallis—together with 95% confidence intervals to aid interpretation of practical significance.

In addition to descriptive and bivariate tests, we performed exploratory multivariate analyses (binary logistic regression) to control for potential confounders such as age, gender, and specialty in selected scenarios where significant group differences were observed.

## 3. Results

### 3.1. Participant Characteristics

A total of 244 respondents completed the survey, comprising 51 senior medical students (20.9%) and 193 practicing clinicians (79.1%). Within the student cohort, the majority were enrolled in Dentistry (82.35%), with 17.65% in General Medicine; the group was predominantly female (84.31%). The physician cohort presented a broader professional spread, with 68.39% dentists and 31.61% general practitioners, and a female proportion of 76.17%. Professional experience among physicians ranged from 1 to 28 years, with a median of 12 years (interquartile range, 6–21), ensuring representation across early-career and senior clinicians. These characteristics are summarized in Table 1. Although the two cohorts differ in size (51 students vs. 193 clinicians), this imbalance reflects the greater accessibility of practicing clinicians compared to the limited pool of final-year students.

### 3.2. Overall Response Patterns to Ethical Dilemmas

Across the questionnaire, respondents most often selected the conditional option-“It depends on the situation”—indicating a strong sensitivity to context in ethical decision-making at both training and practice stages. At the aggregate level, the modal choice was “It depends” (47.8%), followed by “Yes” (33.5%) and “No” (18.7%). This pattern was broadly similar in the two cohorts, with students showing a slightly higher propensity for affirmative responses than physicians (35.6% vs. 32.9%) and physicians selecting “No” marginally more frequently than students (19.4% vs. 16.7%). Taken together, these distributions suggest that respondents commonly weighed situational qualifiers before endorsing a firm action, especially in scenarios with competing principles or legal/organizational constraints.

The prominence of conditional responding was particularly visible in items that foreground value trade-offs. For example, in Item 1 (minimizing risks to obtain consent), “It depends” was chosen by 39.22% of students and 36.27% of physicians (χ^2^ = 0.15, *p* = 0.927), reflecting deliberation over how disclosure, beneficence, and respect for autonomy intersect in practice. Similarly, in a resource-allocation context (Item 26), conditionality again predominated (students 41.18%; physicians 41.97%; χ^2^ test n.s.), consistent with the ethical complexity of distributive judgments when multiple fairness criteria compete.

By contrast, conditional responses tapered in scenarios with clearer professional and legal norms. Items involving the communication of harmful medical errors elicited a strong “No” to concealment in both cohorts, while questions anchored in public health obligations tended to draw decisive responses (e.g., high willingness to treat families who refuse vaccination). These contrasts underscore a coherent pattern: when duties are explicit and widely internalized, respondents converge on categorical choices; when dilemmas hinge on competing principles or scarce resources, respondents default more often to conditional, case-specific reasoning.

Aggregate ‘overall distribution’ was computed by pooling responses across all 35 items (equal weight per item) for each cohort and overall (Table 2).

### 3.3. Comparative Analysis of Ethical Domains

Taken together, the seven ethical domains reveal a shared backbone of professional norms alongside domain-specific nuances where the two cohorts reason differently. Below, we describe the most salient patterns, keeping close to the item-level evidence that underpins each conclusion.

#### 3.3.1. Informed Consent and Transparency

**Figure 1:**
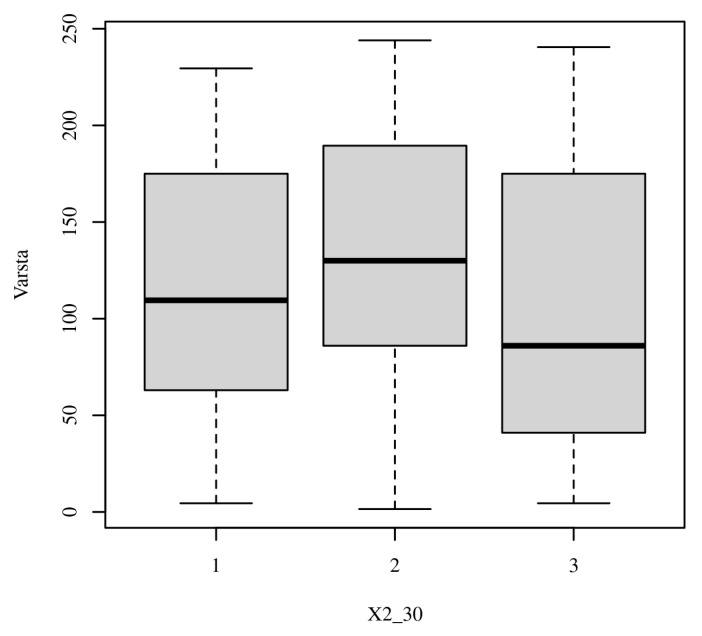
Distribution of responses for Item 29—Comparative boxplot illustrating variability in responses across participant groups for the ethical scenario related to professional responsibility.

When confronted with requests from family members to withhold a diagnosis (Item 3), students were less willing than physicians to comply (i.e., lower “Yes” to withholding: 15.69% vs. 22.80%; χ^2^ = 4.92, *p* = 0.085, Cramer’s V = 0.14, 95% CI [0.05–0.27]). In both groups, however, pluralities rejected nondisclosure outright (students 58.82% “No”; physicians 41.45% “No”), while the remainder selected “It depends,” signaling case-by-case deliberation. These distributions indicate a trend—albeit not statistically significant-toward a stronger disclosure preference among students (see Figure 1).

Attitudes toward error communication show a similar pattern of alignment with professional transparency norms. For harmless errors (Item 4), majorities in both cohorts rejected concealment (students 56.86%; physicians 52.33%; χ^2^ = 0.86, *p* = 0.649, Cramer’s V = 0.06, 95% CI [0.02–0.20]), and for errors with potential harm (Item 5) rejection was near-consensus (students 94.12%; physicians 87.56%; χ^2^ = 1.81, *p* = 0.405, Cramer’s V = 0.09, 95% CI [0.02–0.17]).

#### 3.3.2. Confidentiality and Mandatory Reporting

Public health-adjacent duties elicited broad agreement. In Item 21 (whether to treat families who refuse recommended vaccination), high majorities in both cohorts answered Yes (students 78.43%; physicians 75.13%; χ^2^ = 1.95, *p* = 0.377, Cramer’s V = 0.09, 95% CI [0.03–0.21]), suggesting that refusal of vaccination does not, in itself, threaten the therapeutic relationship.

#### 3.3.3. Medical Error and Professional Responsibility

The paired items on harmless and harmful errors (Items 4–5) reinforce a shared normative stance against concealment. While students consistently trended slightly more toward disclosure, no student–physician contrast reached significance, underscoring that accountability around error communication is widely internalized across career stages.

#### 3.3.4. Preventive Duties and Vaccination

A clearer divergence emerged for occupational vaccination. In Item 22, students more often endorsed annual influenza vaccination for clinicians than physicians (Yes 52.94% vs. 32.64%; χ^2^ = 7.16, *p* = 0.028, Cramer’s V = 0.17, 95% CI [0.07–0.31]), whereas physicians were more frequently opposed (No 40.93% vs. 29.41% among students). The pattern suggests stronger endorsement of prevention-as-duty among trainees.

At a system-responsibility level, Item 20 asked whether individuals with unhealthy lifestyles should pay higher insurance contributions. Here, physicians expressed greater support than students (Yes 48.70% vs. 23.53%; χ^2^ = 11.63, *p* = 0.003, Cramer’s V = 0.22, 95% CI [0.11–0.35]), consistent with a more pragmatic orientation toward sustainability and cost-sharing mechanisms.

#### 3.3.5. End-of-Life Decisions and Resource Allocation

In resource scarcity (Item 26), endorsement of prioritizing younger over older patients was comparable across groups (Yes 35.29% students; 33.68% physicians), with “It depends” attracting the largest share of responses in both cohorts (41.18% vs. 41.97%; χ^2^ = 0.05, *p* = 0.976, Cramer’s V = 0.01, 95% CI [0.02–0.17]). This distribution highlights the moral ambiguity of age-based prioritization and the weight respondents placed on contextual qualifiers.

For euthanasia in incurable illness (additional end-of-life item), overall support was low in both cohorts (students 9.80%; physicians 17.62%), and the difference was not significant; most respondents opposed authorization, with a sizeable minority remaining conditional/undecided.

#### 3.3.6. Conflicts of Interest

Acceptance of benefits linked to medical acts was minimal in both groups (≈5.9% students; 3.63% physicians), and over 87% rejected such practices. The residual “It depends” responses suggest that a small subset still considers context (e.g., nature and magnitude of the benefit), but the overall pattern indicates strong alignment with conflict-of-interest safeguards.

#### 3.3.7. Moral and Cultural Norms

Contrary to a notion of perfect unanimity, responses to romantic/sexual relationships with patients showed clear majority opposition but not total consensus: 56.86% of students and 56.48% of physicians answered No, while 7.84% and 11.92%, respectively, answered Yes, and roughly a third selected “It depends” (χ^2^ = 0.78, *p* = 0.679, Cramer’s V = 0.06, 95% CI [0.02–0.19]).

A related boundary scenario—relationships with a patient’s family member (Item 28)—drew majority rejection as well (No ≈ 51–53%), with ~31% conditional and ~16–18% accepting, again without a significant cohort difference. Together, these items confirm that professional boundaries are widely recognized, while acknowledging lingering ambivalence in a minority.

#### 3.3.8. Ethics in Exceptional Circumstances (Crisis Situations)

Under PPE shortages (Item 34), 43.14% of students and 35.23% of physicians judged abandonment of care as justified, whereas 33.33% and 40.93% opposed it (χ^2^ = 1.28, *p* = 0.528, Cramer’s V = 0.07, 95% CI [0.02–0.21]). When effective treatments/equipment are unavailable (Item 35), ~20% in each group endorsed abandonment (students 21.57%; physicians 19.17%), and majorities opposed (58.82% vs. 55.44%; χ^2^ = 0.76, *p* = 0.685, Cramer’s V = 0.06, 95% CI [0.02–0.19]). These distributions depict a cautious posture in crisis ethics, with meaningful minorities willing to condition the duty to treat on minimal safety/efficacy thresholds.

**Figure 2:**
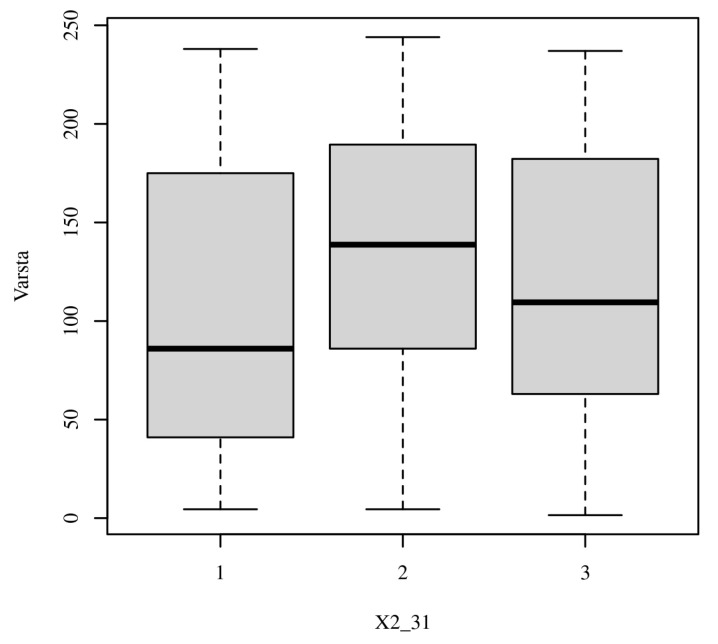
Distribution of responses for Item 31—Comparative boxplot showing the spread and central tendency of responses across cohorts for the ethical scenario addressing confidentiality and professional judgment.

For organ-policy attitudes (Item 24), students expressed greater support than physicians for authorizing the purchase of organs in Romania (Yes 76.47% vs. 62.18%; *p* = 0.043, Cramer’s V = 0.16, 95% CI [0.08–0.25]), pointing to cohort differences in perceptions of policy tools to expand the donor pool (Table 3 and Figure 2).

### 3.4. Summary of Consensus and Divergence

Areas of high consensus. Across cohorts, respondents converged on several core norms. Both students and physicians endorsed continuity of care for families refusing vaccination (Item 21), indicating that vaccine hesitancy, per se, does not warrant limiting access to services. Likewise, acceptance of benefits tied to medical acts was rare in both groups, reflecting strong alignment with conflict-of-interest safeguards. Finally, there was broad rejection of concealing medical errors, with near-uniform opposition when the error had potential for harm, consistent with professional accountability standards.

Areas of divergence. Meaningful differences emerged on issues that straddle personal duty and system stewardship. Students were more supportive of annual influenza vaccination for clinicians (Item 22; χ^2^ = 7.16, *p* = 0.028, V = 0.17, 95% CI [0.07–0.31]), suggesting a stronger endorsement of prevention as a professional obligation. Students also expressed greater support for authorizing the purchase of organs in Romania (Item 24; χ^2^ = 6.32, *p* = 0.043), pointing to differing views on policy instruments to expand the donor pool. In crisis–ethics scenarios involving PPE scarcity (Item 34), the cohorts exhibited modestly different leanings—with students more often judging abandonment of care as justified—although this contrast did not reach statistical significance (χ^2^ = 1.28, *p* = 0.528). Collectively, these patterns indicate that divergences are most likely where ethical judgment must reconcile individual professional duties with broader public health or system-level considerations. The stronger support for annual influenza vaccination among students compared with physicians suggests that preventive duties are more readily endorsed in training contexts. This finding supports considering vaccination policies as a potential option for reinforcing professional responsibility, rather than as a categorical imperative.

In multivariate models, gender and specialty did not consistently predict response patterns, while age showed a modest influence on selected scenarios. These analyses confirmed that the main differences between students and physicians reported above remained robust after adjustment for potential confounders.

## 4. Discussion

This study compared the ethical attitudes of senior medical students and practicing clinicians in Romania underexplored Eastern European context-and identified a pattern of broad agreement on core professional norms alongside targeted divergences with implications for education, policy, and everyday clinical practice. Some findings mirror international trends, whereas others appear grounded in local legal frameworks, modes of professional socialization, and system-level constraints.

Across disclosure scenarios, students showed a tendency toward greater transparency, consistent with international evidence that trainees endorse openness more strongly than senior clinicians [24,25,26,27,28]. This reflects curricular emphasis on autonomy and shared decision-making [29,30], while physicians’ caution may stem from medico-legal exposure and institutional pressures [31,32,33]. Pedagogically, these findings support structured training in error disclosure to sustain transparency during the transition to independent practice.

The high frequency of “It depends” answers in our data reflects a context-sensitive approach to ethical judgment. This tendency resonates with conditional moral reasoning frameworks, which emphasize that individuals often balance competing principles (e.g., autonomy vs. beneficence) rather than applying categorical rules. Prior research on cognitive framing suggests that situational qualifiers activate deliberative processes, prompting respondents to consider contextual factors such as legal constraints, institutional policies, or potential harm before endorsing a definitive action. Such findings align with theories of moral particularism, where ethical reasoning is guided less by rigid rules and more by the perceived salience of context-specific details. In the medical domain, this pattern underscores how both trainees and practitioners negotiate uncertainty by appealing to situational contingencies rather than universal imperatives. This interpretation is consistent with recent cross-cultural analyses showing that moral framing among healthcare trainees is strongly shaped by cultural and institutional contexts [34].

Both cohorts showed strong consensus on public health obligations such as mandatory reporting, aligning with European findings [35,36,37,38] and reflecting duties codified by law and policy. Physicians’ hesitancy around harmless-error disclosure, often linked to fear of sanctions and mistrust [9,39,40,41], contrasts with students’ principle-driven stance and underscores the need for institutional supports such as clear protocols and legal safe harbors.

Students showed a tendency toward euthanasia in terminal illness, a trend consistent with other European surveys [21,42,43,44,45,46]. Physicians’ lower acceptance likely reflects Romania’s legal prohibitions and clinical realities, underscoring the need to integrate both palliative frameworks and legal literacy into ethics education [44,45]. It should be noted, however, that while end-of-life scenarios are highly pertinent to general medical practice, their direct applicability to dental practice is more limited. Nevertheless, their inclusion enabled comparative insight into how different professional cohorts, medical and dental, approach ethically complex issues, even when these extend beyond their immediate scope of practice.

Our instrument included a scenario on accepting non-monetary benefits from pharmaceutical representatives, which allowed us to capture attitudes toward conflicts of interest. The low acceptance rates observed are consistent with international findings that even small gifts may bias judgment and erode public trust. Nevertheless, international literature indicates that acceptance of industry interactions tends to rise with practice exposure [23,47,48], a dynamic sometimes described as normalization [49]. Professional codes and institutional policies caution that even small gifts may bias judgment and erode public trust [50,51]. Future iterations of the survey should include explicit scenarios on industry relations to enable granular trainee–clinician comparisons.

A minority tolerated romantic or sexual relationships with patients, despite clear international prohibitions [52]. In crisis situations (e.g., PPE shortages), both groups showed cautious conditional reasoning, consistent with utilitarian allocation principles in pandemic ethics frameworks [53,54,55]. Moreover, emerging pharmacological perspectives suggest that decision-making under uncertainty engages both cognitive and affective processes, reinforcing the importance of situational framing in ethical judgment [56].

Significant divergences appeared where individual duties intersected with system stewardship: (i) Physicians were more supportive of higher insurance contributions for unhealthy lifestyles, consistent with a system-stewardship perspective shaped by long-term exposure to financing and prevention incentives. (ii) Students more often endorsed annual influenza vaccination for clinicians in patient-facing roles, aligning with a training environment that foregrounds population-level protection. Together, these contrasts suggest that experiential factors recalibrate ethical judgments in domains that couple personal duty with system-level considerations.

This study has several limitations. The findings are culturally specific to Romania and may not be directly generalizable to other contexts. The use of hypothetical scenarios may not fully capture the complexity of real-life decision-making, and reliance on self-reported responses introduces potential social desirability and recall biases. Variability in age, training background, and clinical specialty may have contributed to some differences, although multivariate analyses indicated only modest age effects. The sample was predominantly female, which may further limit generalizability to more gender-balanced populations. The trainee cohort was largely composed of dental students, which may reduce comparability with physicians trained in general medicine. Although dentistry and general medicine differ in scope, Romanian legislation classifies both as branches of medicine, which justifies their combined analysis; nevertheless, not all 35 scenarios are equally relevant across professions. Despite these constraints, the consistency of patterns across subgroups and the robustness of multivariate results support the validity of our conclusions.

The findings support a two-pronged strategy. First, undergraduate programs should enrich principlist content with scenario-based simulations and longitudinal mentorship to bridge the theory–practice gap [57,58]. Second, continuing professional development (CPD) for physicians should reinforce patient-centered transparency, strengthen skills in communicating uncertainty and errors, and offer structured guidance on conflicts of interest and end-of-life decisions [59,60]. At the institutional level, clear, supportive error-disclosure protocols and explicit policies on industry relations may reduce defensive behavior and unwarranted variability. By situating these dynamics in an Eastern European context, our study offers actionable directions for aligning ethics education with clinical realities and, ultimately, for strengthening trust, fairness, and integrity in patient care.

In interpreting these findings, it is important to recognize that dentistry, while legally classified as a branch of medicine in Romania, presents its own distinctive ethical challenges. Issues such as informed consent in elective procedures, error disclosure in restorative and surgical contexts, and conflicts of interest with industry representatives are increasingly highlighted in the literature [10,61]. The strong alignment observed across both medical and dental respondents in our study therefore underscores shared professional norms, while also pointing to areas where dental ethics frameworks may offer complementary insights.

## 5. Conclusions

This exploratory study highlights both consensus and divergence in ethical reasoning between Romanian medical students and practicing clinicians. While the findings suggest implications for ethics education and institutional policies—such as supportive error-disclosure protocols and reinforcement of preventive duties—they should be interpreted with caution given the cultural specificity, reliance on hypothetical scenarios, and self-report design. Rather than definitive prescriptions, the results provide indicative directions for strengthening transparency, stewardship, and patient-centered care, and they call for replication in broader and more diverse samples.

At the policy level, clear error-disclosure protocols, explicit guidance on conflicts of interest and boundary setting, and transparent crisis–ethics frameworks can reduce defensive behavior and unwarranted variability. In line with our findings, measures such as continuing professional development in ethical communication and the possible introduction of annual influenza vaccination policies for healthcare personnel should be considered as policy options, rather than categorical imperatives. Grounded in an Eastern European context that is underrepresented in the literature, these results offer evidence-informed proposals for aligning ethics education with clinical realities and strengthening trust, fairness, and integrity in patient care.

## Figures and Tables

**Table 1:** Demographic and professional characteristics of study participants.

Variable	Students (*n* = 51)	Physicians (*n* = 193)	Total (*n* = 244)
Gender—male, *n* (%)	8 (15.69)	46 (23.83)	54 (22.13)
Gender—female, *n* (%)	43 (84.31)	147 (76.17)	190 (77.87)
Specialization—Dentistry, *n* (%)	42 (82.35)	132 (68.39)	174 (71.31)
Specialization—General Medicine, *n* (%)	9 (17.65)	61 (31.61)	70 (28.69)
Median years of experience (IQR)	-	12 (6–21)	-

**Table 2:** Overall distribution of responses across all participants.

Response Option	Students (%)	Physicians (%)	Total (%)
Yes	35.6	32.9	33.5
No	16.7	19.4	18.7
It Depends	47.7	47.7	47.8

**Table 3:** Selected Ethical Scenarios.

Item	Scenario	Students: Yes (%)	Students: No (%)	Students: It Depends (%)	Physicians: Yes (%)	Physicians: No (%)	Physicians: It Depends (%)	*p*-Value
Item 3	Withhold diagnosis on family request	15.69	58.82	25.49	22.8	41.45	35.75	0.085
Item 4	Hide medical error (no harm)	23.53	56.86	19.61	21.76	52.33	25.91	0.649
Item 5	Hide medical error (with harm)	1.96	94.12	3.92	5.18	87.56	7.25	0.405
Item 20	Higher insurance for unhealthy lifestyle	23.53	49.02	27.45	48.7	37.31	13.99	0.003
Item 21	Treat families refusing vaccines	78.43	5.88	15.69	75.13	12.44	12.44	0.377
Item 22	Annual influenza vaccine for clinicians	52.94	29.41	17.65	32.64	40.93	26.42	0.028
Item 24	Authorize purchase of organs (RO)	76.47	5.88	17.65	62.18	20.73	17.1	0.043
Item 26	Prioritize younger patients (scarcity)	35.29	23.53	41.18	33.68	24.35	41.97	0.145
Item 34	Abandon care if PPE insufficient	43.14	33.33	23.53	35.23	40.93	23.83	0.528
Item 35	Abandon care if no treatments available	21.57	58.82	19.61	19.17	55.44	25.39	0.685

## Data Availability

The data presented in this study are available on request from the corresponding author due to privacy and ethical restrictions.

## Abbreviations

The following abbreviations are used in this manuscript:

CPD	Continuing Professional Development
IRB	Institutional Review Board
PPE	Personal Protective Equipment
WMA	World Medical Association
χ^2^	Chi-square
